# Current situation of scrub typhus in South Korea from 2001–2013

**DOI:** 10.1186/s13071-015-0858-6

**Published:** 2015-04-19

**Authors:** Hyeong-Woo Lee, Pyo Yun Cho, Sung-Ung Moon, Byoung-Kuk Na, Yoon-Joong Kang, Youngjoo Sohn, Seung-Ki Youn, Yeongseon Hong, Tong-Soo Kim

**Affiliations:** Department of Pathology, Immunology, and Laboratory Medicine, College of Medicine, University of Florida, Gainesville, FL 32610 USA; Departments of Parasitology and Tropical Medicine, Inha University School of Medicine, Incheon, 400-712 Republic of Korea; Department of Internal Medicine, Seoul National University Bundang Hospital, Seongnam, 463-939 Korea; Department of Parasitology and Institute of Health Sciences, Gyeongsang National University School of Medicine, Jinju, 660-751 Korea; Department of Biomedical Science, Jungwon University, Goesan, Chungbuk 367-805 Republic of Korea; Department of Anatomy, College of Korean Medicine, Institute of Korean Medicine, Kyung Hee University, Seoul, 130-701 Korea; Department of Epidemiology, National Institute of Health, Osong, 363-951 Republic of Korea; Department of Public Health, Sahmyook University, Seoul, 139-742 Republic of Korea

**Keywords:** Female, Incidence, Mites, *Orientia tsutsugamushi*, Republic of Korea, Scrub typhus, Trombiculidae

## Abstract

**Background:**

The bacteria *Orientia tsutsugamushi* is the causative agent of scrub typhus, mite-borne disease, which causes an acute febrile illness in patients. An epidemiologic study was conducted to understand the characteristics of scrub typhus in South Korea.

**Findings:**

Reporting of tsutsugamushi disease is mandatory in South Korea since 1994. To investigate the prevalence of tsutsugamushi disease from 2001 to 2013, medical records from the Korea Center for Disease Control and Prevention were reviewed. In total, 70,914 cases were reported during 2001–2013. Of these, 37.16% (26,349) were male and 62.84% (44,565) were female. The highest number of cases was in the 60–69-year-old age group (19,484; 27.48%), and 72.22% (51,212) were in the 50–79-year-old age group. There were 65,100 cases (91.80%) reported during October (24,964; 35.20%) and November (40,136; 56.60%). An almost four-fold increase in the number of patients was observed in 2013 (10,485 cases) compared to 2001 (2,637 cases). The highest number of patients was reported in the Jeonbuk (9,425; 13.29%) and lowest in the Jeju (362; 0.51%).

**Conclusions:**

A rapid increase in the incidence of patients with tsutsugamushi disease was observed in most areas from 2001 to 2013, with the majority of cases reported in the western and southern coast.

## Findings

### Background

Scrub typhus, tsutsugamushi disease, or chigger-borne rickettsiosis is an acute, febrile infectious disease among humans that is caused by infection with the bacterium *Orientia tsutsugamushi* following the bite of infected mite vectors. It is prevalent in the Asia-Pacific region, where about 1 million cases are reported annually and about 1 billion people may be at risk [1,2]. In South Korea, scrub typhus was first reported in six cases during the Korean War among United Nations military personnel [3], however, tsutsugamushi disease was unfamiliar to Koreans until 1986 when some suspected patients were diagnosed with tsutsugamushi disease. After that, scrub typhus was recognized as endemic in South Korea [4,5], and since then, the disease has been one of the most common rickettsial diseases with poorly understood reasons and then become a major public health problem to farmers during harvest season. In 1994, scrub typhus was designated a Group III notifiable infectious disease by the Korea Centers for Disease Control and Prevention (KCDC). Subsequently, approximately 300 cases were reported annually, with a peak incidence of 6,562 cases in 2005 and a plateau pattern thereafter [6,7]. Since 2012, however, disease incidence has rapidly increased. Here, we report on the epidemiology of scrub typhus in South Korea in the last decade in order to enhance understanding of its geographic, temporal and demographic characteristics.

### Methods

Data from the National Infectious Disease Surveillance (NIDS) system were used to analyze the epidemiology of scrub typhus in South Korea. The geographic distribution of the disease was determined based on patient residence at the time of diagnosis. The annual prevalence rate (APR: the number of cases per 100,000 people) was used to further analyze its geographic distribution. The disease’s seasonal incidence was determined by grouping cases in monthly intervals. After obtaining approval from the Institutional Review Board of the Korean National Institute of Health, scrub typhus case records submitted to the KCDC were reviewed without revealing patient identity, and trends based on patients’ age, sex, and occupation were analyzed.

### Results and discussion

**Figure 1:**
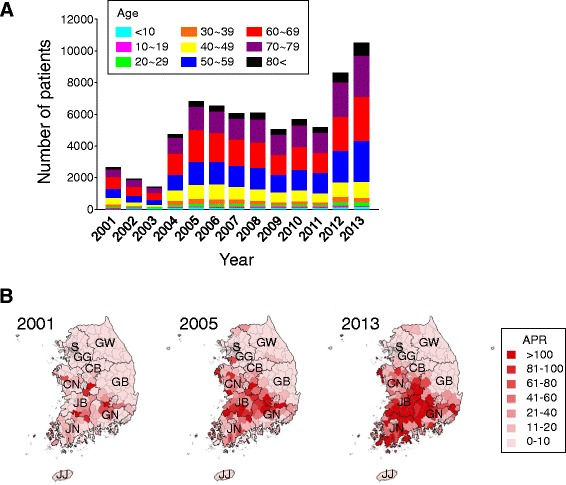
Scrub typhus in South Korea. **(A)** Total incidence of scrub typhus in South Korea between 2001–2013. **(B)** Annual prevalence rate and space-time clustering of scrub typhus cases at the county level in South Korea. SE, Seoul; GG, Gyeonggi Province; GW, Gangwon Province; CB, Chungbuk Province; CN, Chungnam Province; JB, Jeonbuk Province; JN, Jeonnam Province; GB, Gyeongbuk Province; GN, Gyeongnam Province; JJ, Jeju Province.

Analysis of the NIDS data revealed 70,914 cases of scrub typhus in South Korea from 2001 to 2013. Fewer than 2,700 cases were reported annually from 2001 to 2003, after which the incidence rapidly rose to a small peak of 6,780 cases in 2005, and then settled into a plateau pattern with a fluctuating number of annual indigenous cases. However, in 2012 and 2013 the incidence rapidly increased, with 10,485 cases reported in 2013 (Figure 1A).

The incidence of scrub typhus varies with sex, age, and occupation. Scrub typhus patients were more likely to be female (44,565 cases, 62.84%) than male (26,349 cases, 37.16%). The sex-specific incidence (female/male ratio) did not change over time, varying slightly between 1.56 and 1.91 per year. Patients aged 60–69 years (19,484 cases) comprised the highest number of cases, followed by those aged 70–79 (16,141 cases) and 50–59 (15,587 cases) years. These age groups comprised 72.2% of the total number of scrub typhus cases (Figure 1A, Table 1). It is worth noting, however, that the disease has been increasing in all age groups, especially in children aged less than 10 years. Farmers had a higher incidence of scrub typhus than non-farmers, although the proportion of cases in non-farmers increased over the study period. The majority (91.8%) of cases occurred in October and November, but a considerable number were also reported in December (3,995 cases, 5.6%). Lowest cases (75 cases, 0.1%) were reported in March. Interestingly, 389 cases (0.5%) were reported between January and February, i.e. the winter season in South Korea (Table 2).

**Table 1:** **The number of Scrub typhus cases according to age in South Korea during 2001-2013**

	**2001**	**2002**	**2003**	**2004**	**2005**	**2006**	**2007**	**2008**	**2009**	**2010**	**2011**	**2012**	**2013**	**Total**
<10	44	38	16	55	71	62	49	45	49	60	57	96	92	734
10 - 19	26	15	20	55	79	69	76	61	64	77	79	124	111	856
20 - 29	76	36	30	105	152	148	147	121	98	133	117	202	226	1,591
30 - 39	168	108	62	297	372	350	320	259	215	250	205	444	379	3,429
40 - 49	407	230	171	661	896	963	795	735	600	670	546	888	983	8,545
50 - 59	556	366	265	928	1,408	1,378	1,281	1,320	1,076	1,259	1,231	1,970	2,549	15,587
60 - 69	760	653	474	1,375	2,022	1,806	1,701	1,662	1,315	1,473	1,308	2,164	2,771	19,484
70 - 79	478	387	295	973	1,419	1,348	1,303	1,454	1,237	1,349	1,241	2,099	2,558	16,141
80 - 89	115	84	80	239	341	332	335	374	321	368	349	584	764	4,286
90 >	7	2	2	10	20	24	15	26	20	32	18	33	52	261
Total	2,637	1,919	1,415	4,698	6,780	6,480	6,022	6,057	4,995	5,671	5,151	8,604	10,485	70,914

**Table 2:** **The number of Scrub typhus cases by month in South Korea during 2001-2013**

	**Jan**	**Feb**	**Mar**	**Apr**	**May**	**Jun**	**Jul**	**Aug**	**Sep**	**Oct**	**Nov**	**Dec**	**Total**
2001	5	1	1	1	1	0	4	4	7	1,007	1,499	107	2,637
2002	6	3	3	1	7	7	3	5	22	962	843	57	1,919
2003	5	3	2	4	7	6	3	1	9	642	642	91	1,415
2004	7	1	2	2	6	6	0	6	14	1,829	2,667	158	4,698
2005	10	2	0	3	4	9	7	5	24	2,159	4,167	390	6,780
2006	11	3	4	13	8	9	5	5	29	2,054	3,766	573	6,480
2007	25	10	6	11	13	17	12	11	26	1,123	4,374	394	6,022
2008	14	10	2	8	22	8	12	6	38	3,035	2,547	355	6,057
2009	39	17	13	15	14	21	14	12	51	1,739	2,641	419	4,995
2010	23	13	11	16	27	27	27	27	60	1,203	3,832	405	5,671
2011	37	11	10	13	25	24	18	28	77	1,825	2,718	365	5,151
2012	51	41	15	17	20	14	25	36	102	3,680	4,341	262	8,604
2013	25	16	6	9	31	33	40	27	74	3,706	6,099	419	10,485
Total	258	131	75	113	185	181	170	173	533	24,964	40,136	3,995	70,914

Cases of scrub typhus were not evenly distributed throughout the country. It showed that the disease spread over time was more prevalent in rural areas than in urban areas. The highest number of cases was reported in Jeonbuk Province (JB, 9,425 cases; APR, 40.23) followed by Jeonnam Province (JN, 7,403 cases; APR, 32.14), Chungnam Province (CN, 8,493 cases; APR, 30.73), and Gyeongnam Province (GB, 8,746 cases; APR, 20.82) (Figure 1B). The primary industry in these provinces is agriculture or agriculture-related activities, and most patients were farmers. Since most cases of scrub typhus occur in elderly farmers, the higher incidence of female patients may be due to differences in work behavior between men and women during the harvest season, when there is an increased possibility of exposure to infected chigger mites. Cases of scrub typhus have also increased in urban and suburban areas, correlating with the increase in the incidence of the disease in non-farmers. For instance, the APR in Seoul Metropolitan City increased from 0.54 in 2003 to 3.75 in 2012, in Busan Metropolitan City from 1.50 in 2003 to 23.30 in 2013, in Daegu Metropolitan City from 1.01 in 2003 to 19.54 in 2005, in Incheon Metropolitan City from 0.25 in 2002 to 4.44 in 2012, in Gwangju Metropolitan City from 3.45 in 2003 to 32.93 in 2013, in Daejeon Metropolitan City from 2.03 in 2003 to 33.86 in 2013, and in Ulsan Metropolitan City from 2.81 in 2003 to 59.73 in 2013. In our previous study about the geographical distribution of scrub typhus vectors, a survey of larval trombiculid mites had been conducted during 2005 to 2007 by collecting wild small twice a year at 24 sites nationwide. The predominant mite species were *Leptotrombidium pallidum* (52.6%), *Leptotrombidium scutellare* (27.1%), *Leptotrombidium palpale* (8.2%), *Leptotrombidium orientale* (5.6%), and *Neotrombicula tamiyai* (1.7%). However, the geographical distribution map of the *L. scutellare* chigger index was identical to the incidence pattern of scrub typhus, whereas those of overall mites and *L. pallidum* showed no relationship with case incidence patterns [8].

The recent expansion of scrub typhus in South Korea may be closely associated with factors such as the physical environment and human activities. Future research is required to identify the role these factors play in the epidemiology of the disease. It is expected that the number of scrub typhus cases will continue to increase, although there may be some fluctuations. An intensive nationwide surveillance system is needed to ensure rapid diagnosis and treatment, and to monitor the changing environment, including the rodent population, and emerging drug resistant strains, until effective vaccine development reduces its incidence. In addition, education of the public through the media on the risk factors and symptoms of scrub typhus, together with personal protection methods during farming, gathering chestnuts, even outdoor activities during winter season, and taking breaks in areas adjacent to agricultural operations, should be undertaken.
